# Hydrojet Surface Treatment of Ti-6Al-4V Titanium Produced by Additive Manufacturing

**DOI:** 10.3390/ma18174150

**Published:** 2025-09-04

**Authors:** Monika Szada-Borzyszkowska, Dorota Laskowska, Błażej Bałasz, Wiesław Szada-Borzyszkowski

**Affiliations:** 1 Faculty of Mechanical Engineering and Energy, Koszalin University of Technology, Śniadeckich 2, 75-620 Koszalin, Poland; blazej.balasz@tu.koszalin.pl; 2 Branch of the KUT in Szczecinek, Koszalin University of Technology, 78-400 Szczecinek, Poland; wieslaw.szada-borzyszkowski@tu.koszalin.pl

**Keywords:** additive manufacturing, Ti-based alloys, water jet, abrasive water jet, water–ice jet

## Abstract

The aim of this study was to analyze the effect of finishing methods on the surface quality of Ti-6Al-4V titanium alloy additively manufactured by selective laser melting. It was observed that among the finishing methods, water jet treatment did not produce significant changes, while the abrasive water jet proved effective in removing defects and smoothing the surface, especially at a pressure of 30 MPa. However, the risk of abrasive particle entrapment in the material was observed. Promising results were also obtained using the water–ice jet, which combines effective material removal with surface smoothing. The selection of the finishing method should be tailored to the application requirements. Further research will focus on optimization and the combination of techniques to improve the functional properties of titanium components.

## 1. Introduction

Surface treatment of Ti-6Al-4V titanium alloy plays a key role in ensuring the high quality of components used in the aerospace [1,2] and medical [3,4] industries. Due to its excellent mechanical properties [5], corrosion resistance, and biocompatibility, Ti-6Al-4V is widely used in the production of components requiring high strength and durability [6].

In recent years, additive manufacturing techniques such as selective laser melting (SLM) [7] have gained popularity, enabling the production of components with complex geometries [8] and optimized mechanical properties [9]. Wei et al. [10] presented an approach combining the adjustment of SLM process parameters with subsequent surface finishing, which made it possible to obtain an optimized microstructure and increased ductility. However, the surfaces of additively manufactured parts are characterized by high roughness [11], which can negatively affect their fatigue properties and overall functionality [12]. Recent studies have also highlighted that surface modifications can significantly influence the electrochemical behavior and corrosion resistance of Ti-6Al-4V alloys, especially under simulated inflammatory conditions, which is critical for medical applications [13].

To improve the surface quality of such components, various finishing methods are applied, including laser polishing [14], abrasive fluidized bed machining [15], chemical polishing [16], and techniques utilizing laser-induced cavitation [17]. Laser polishing, using pulsed or continuous lasers, enables a significant reduction in surface roughness and an improvement in wear and corrosion resistance, without the need for chemicals or mechanical tools [18]. Abrasive fluidized bed machining involves the impact of abrasive particles on the surface of the workpiece, resulting in surface smoothing and the introduction of compressive stresses that positively affect fatigue life [15]. In contrast, chemical polishing, although effective in reducing roughness, requires the use of aggressive and hazardous chemicals such as hydrofluoric acid [16]. Methods based on laser-induced cavitation [17] have demonstrated potential to enhance fatigue properties and reduce microporosity.

Shinonaga T. et al. [19] investigated an integrated surface smoothing method for additively manufactured Ti-6Al-4V titanium alloy, which combines sandblasting with irradiation by a large electron beam (LEB). The combination of these techniques enables a significant reduction in surface roughness (Rz, from approximately 265 µm to about 2 µm) and improves mechanical properties through the induction of compressive stresses. The study demonstrated that prior sandblasting enhances the effectiveness of LEB smoothing by reducing the spacing of roughness peaks. This method represents a promising solution for improving the surface quality of Ti-6Al-4V components in industrial and biomedical applications.

It should be emphasized that surface contamination and topography have direct biomedical implications. Residual abrasive or metallic particles may trigger local inflammatory responses and bone resorption, compromising implant stability [20,21]. Moreover, surface roughness strongly influences osseointegration: excessive roughness can promote bacterial adhesion and peri-implant infection, whereas overly smooth surfaces may hinder osteoblast attachment and proliferation [22]. Therefore, optimization of surface finishing methods is critical to balance the mechanical performance and biological compatibility of Ti-6Al-4V implants.

Studies conducted by Żebrowski R. et al. [23] demonstrated that shot peening can beneficially influence the corrosion resistance of Ti-6Al-4V alloy produced by the DMLS method. Analysis using three media and various pressures reveals that the improvement mainly results from grain refinement and accelerated formation of the passive layer. Excessively high peening pressure increased surface roughness and deteriorated anti-corrosion properties. Properly selected process parameters can significantly enhance the material’s suitability for medical applications.

Santhosha Rathnam G. et al. [24] analyzed the influence of different abrasive materials (such as Al_2_O_3_, glass, garnet sand, and Al metal grits) used in shot blasting on the surface quality of Ti-6Al-4V (Grade 5) titanium alloy prior to the coating process. The study showed that abrasive treatment significantly affects surface roughness, cleanliness, and microstructure, which are critical for the adhesion of protective coatings such as PVD. In particular, it was indicated that Al_2_O_3_ grains are effective but may also cause surface contamination. The selection of appropriate process parameters and abrasive types is essential to achieve optimal surface properties of finished titanium components.

Okan et al. [25] focused on analyzing the influence of blasting parameters (garnet grain size and stream pressure) on the surface topography and roughness of the Ti-6Al-4V alloy. The research demonstrated that variations in these parameters significantly affect the surface structure, which is of considerable importance in the context of substrate preparation for protective coatings such as PVD.

Fuse et al. [26] and Bound et al. [27] conducted studies on abrasive jet machining of the Ti-6Al-4V titanium alloy to optimize process parameters. The results showed that key process parameters, such as nozzle traverse speed, abrasive flow rate, and the distance between the nozzle and the workpiece, have a significant effect on the material removal rate, surface roughness, and taper angle. The findings indicate that process parameters substantially influence the efficiency of abrasive jet machining of titanium alloys, which is relevant in the context of the industrial application of this technology.

In the study by Soyama et al. [28], a technique combining abrasive blasting with cavitation peening was proposed, which enables the simultaneous introduction of compressive stresses and reduction of surface roughness. The study demonstrated that the application of this method improves the fatigue strength of the material from 169 MPa to 280 MPa. The results suggest that combining these two processes can be an effective strategy for enhancing the mechanical properties of additively manufactured titanium components.

It is worth highlighting the studies conducted by Rauch et al. [29], in which four surface finishing methods for additively manufactured Ti-6Al-4V alloy were analyzed—sandblasting, electropolishing, chemical polishing, and abrasive flow polishing. Sandblasting contributes to the reduction of surface roughness with minimal material loss, although it may leave abrasive residues (corundum). Chemical polishing provides the best smoothing effect but is more aggressive. Abrasive flow polishing is particularly effective for internal surfaces of channels, efficiently eliminating geometric defects. The study suggests that a combination of these technologies—e.g., sandblasting, chemical polishing, and abrasive flow polishing—can optimize surface quality and the functionality of finished components.

The aim of this study was to evaluate the effectiveness of using a high-pressure water jet and a water jet combined with selected abrasives, such as quartz sand and dry ice (CO_2_). The analysis was conducted in the context of improving surface quality and the efficiency of treatment processes for components additively manufactured by selective laser melting (SLM) from Ti-6Al-4V powder. The main objective was to compare the effectiveness of the discussed methods in reducing surface roughness and removing residual powder and other contaminants. These issues are crucial for ensuring the appropriate mechanical and functional properties of the final components. The scientific objective of this work was to elucidate the effects of high-pressure water jet and abrasive-assisted treatments on the surface integrity of Ti-6Al-4V components fabricated by selective laser melting. Particular attention was given to the mechanisms of surface roughness reduction and the removal of residual contaminants. The study further aimed to clarify how these post-processing methods influence the functional performance of additively manufactured parts.

## 2. Materials and Methods

### 2.1. Samples Preparation

The 10 × 10 × 10 mm samples were produced using selective laser melting (SLM) technology with an ORLAS CREATOR^®^ 3D printer (O. R. Lasertechnologie GmbH, Dieburg, Germany). The process parameters were selected based on the research conducted by Laskowska et al. [30], which demonstrated that surface roughness of components manufactured using the SLM technology can be controlled to a limited extent by adjusting process parameters. Based on this, two manufacturing strategies were selected, as presented in Table 1. In the study by Laskowska et al. [30], samples produced using the S5 strategy exhibited the highest surface roughness (Sa = 26.2 ± 5.2 μm), whereas those produced with the S8 strategy showed the lowest roughness (Sa = 10 ± 3.6 μm).

Commercially available Ti-6Al-4V titanium alloy powder (3D Systems, Rock Hill, SC, USA) was used for sample fabrication. The chemical composition of the powder is presented in Table 2. Post-processing of the samples included mechanical removal of support structures and ultrasonic cleaning in distilled water for 10 min.

### 2.2. Experimental Setup for Surface Treatment

Surface treatment of additively manufactured Ti-6Al-4V alloy samples were carried out using three methods: high-pressure water jet, abrasive water jet, and water–ice jet. The high-pressure water jet technique uses a concentrated stream of water under high pressure to remove material from metallic surfaces. Due to its effectiveness and safety, it is suitable for the treatment of delicate components. The treatment process using an abrasive water jet involves abrasive particle mixtures (such as quartz sand) suspended in water. This method enables a more aggressive surface treatment, making it suitable for applications requiring higher surface quality and allowing for the removal of greater amounts of material. The water–ice jet technique combines dry ice with water to achieve effective surface treatment while minimizing the risk of overheating and deformation. This is especially critical when processing titanium components.

**Figure 1:**
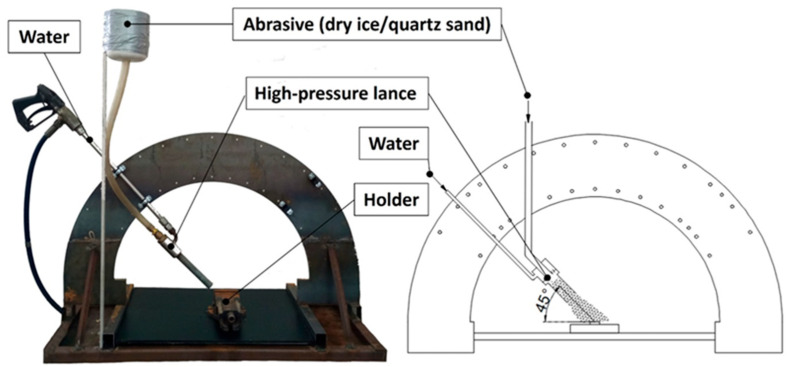
Experimental setup for surface treatment.

The surface treatments were conducted at various water pressures (generated at the outlet of the high-pressure pump): 10 MPa, 20 MPa, and 30 MPa. In all cases, the jet was applied to the surface for the same duration of 15 s. To perform experiments, a dedicated experimental setup was designed (Figure 1).

The study employed a high-pressure lance with a four-orifice nozzle, each with a diameter of 0.6 mm. Due to the mechanical properties of titanium and the resulting optimal processing efficiency, the nozzle angle was set to 45° [31]. The distance between the jet outlet and the treated surface was maintained at a constant 50 mm during all experiments. This design ensured a uniform and favorable distribution of abrasive particles within the high-pressure jet. The precise formation and focusing of the individual water streams were critical for achieving effective surface treatment of titanium.

The process was carried out using a WEMAA hydro monitor (Annovi Reverberi, Modena, Italy) with an electric drive, equipped with an As500/15A-type pump. Detailed specifications of the device are provided in Table 3.

### 2.3. Abrasive Materials

**Figure 2:**
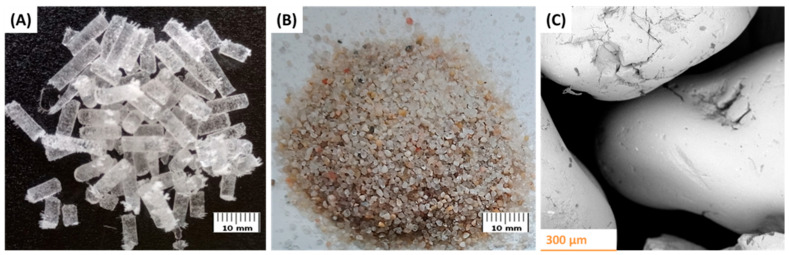
Abrasive materials used in the surface treatment process: (**A**) dry ice (CO_2_) granules, (**B**) quartz sand. (**C**) SEM image of quartz sand.

The abrasive materials used in the surface treatment process were quartz sand and dry ice (CO_2_) granules (Figure 2). The specifications of both materials are presented in Table 4.

SEM characterization of the quartz sand particles (Figure 2C) revealed a predominantly rounded shape, which is expected to influence their mechanical interaction with the substrate and affect the extent and nature of particle embedment during the surface treatment process.

Quartz sand is a variety of coarse-crystalline silica (SiO_2_) commonly used as an abrasive material in abrasive water jet technologies. Dry ice sublimates without transitioning into the liquid phase, thereby eliminating the risk of introducing moisture into the treated area. In the present study, its use is associated with its ability to effectively remove contaminants without leaving abrasive residue on the surface. This property is particularly important in the treatment of materials with high surface reactivity, such as titanium, where maintaining a high level of surface cleanliness is crucial for subsequent analysis or operational performance.

### 2.4. Analysis of Surface Morphology and Topography

To evaluate the surface topography before and after treatment with water, abrasive water, and water–ice jets, a 3D optical microscope InfiniteFocus G6 (Bruker Alicona, Alicona Imaging GmbH, Graz, Austria) was used. The device facilitated a detailed analysis of surface roughness, including parameters such as arithmetical mean height (Sa), maximum profile height (Sz), root mean square height (Sq), and maximum pit depth (Sv).

Surface morphology analysis before and after treatment was conducted using a scanning electron microscope Phenom ProX, (Phenom-World BV, Eindhoven, The Netherlands). This device allowed for high-resolution observation of residual processing effects on the surface of Ti-6Al-4V titanium alloy.

## 3. Results and Discussion

### 3.1. Characteristics of the Surface of As-Built Samples

**Figure 3:**
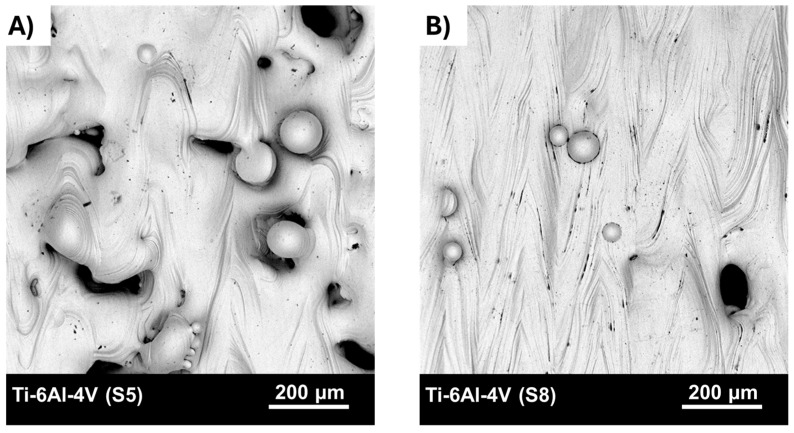
SEM images of top surface for: (**A**) Ti-6Al-4V (S5) samples, (**B**) Ti-6Al-4V (S8) samples.

The investigation began with the characterization of the surface of as-built Ti-6Al-4V alloy samples produced by selective laser melting technology. Figure 3 shows representative SEM micrographs obtained from randomly selected regions of the top surfaces of the examined specimens.

The top surface of the samples produced using strategy S5 exhibited a high degree of surface irregularity. A considerable number of defects were observed along the laser scan tracks, which is typical for processing conditions involving high scan speed and low laser power. Under such conditions, insufficient melting of the powder bed occurs, leading to the formation of lack-of-fusion defects within the manufactured part. On the top surface, numerous open surface pores (craters) were detected, likely extending several layers deep into the component. These pores may entrap unmelted or partially fused powder particles. Furthermore, such defects may act as pathways for the ingress of water or contaminants into the interior of the part.

The condition of the top surface of the samples fabricated using strategy S8 indicates that the additive manufacturing process was carried out under stable conditions, known as conduction mode. In some areas, surface irregularities and depressions between adjacent laser scan tracks were observed. Characteristic ripples were visible along the scan paths, resulting from the Marangoni effect—a thermocapillary flow of the molten material caused by gradients in temperature and surface tension within the melt pool. The presence of surface pores was significantly reduced compared to strategy S5; the pores observed were more regularly shaped and smaller in size.

Additionally, partially melted powder particles were observed on the top surfaces of both samples. This phenomenon can be attributed to thermal diffusion or the so-called spatter effect. In the former case, unmelted powder particles are attracted due to surface tension forces. The high temperature of the melt pool causes these particles to partially melt and adhere to the edge of the pool.

In the case of the spatter effect, the high temperature of the melt pool causes the evaporation of alloying elements. The resulting recoil pressure acts on the melt pool, ejecting molten material in the form of a jet, which subsequently breaks up into microdroplets (droplet spatter). This pressure also causes the dispersion of unmelted powder particles located at the edges of the melt pool (lateral spatter). The size and morphology of the particles are preserved. The products of both droplet and lateral spatter can deposit on the originating component or surrounding parts, influencing the microstructure and surface roughness of the top surface. Studies by Liu et al. [32] indicate that the size, dispersion state, and height of the spatter depend on the laser power.

Studies show that the surface condition after the additive manufacturing process using SLM technology depends on the stability of the material melting process. However, despite the partial elimination of surface defects through modification of key process parameters, the surface roughness averaged approximately 30 μm for Ti-6Al-4V (S5) and around 17 μm for Ti-6Al-4V (S8). Such high surface roughness of additively manufactured components may significantly limit their practical applications. Control of surface roughness solely by adjusting process parameters appears to be limited; therefore, the implementation of post-processing surface finishing should be considered.

### 3.2. Characteristics of the Surface After High-Pressure Water Jet Treatment

As a first step, the additively manufactured titanium alloy samples were treated using a water jet. To evaluate surface morphology and roughness, non-contact measurement methods were used. These measurements enable the determination of relationships between surface geometry and surface geometric parameters (SGPs) in relation to the processing conditions, which may significantly affect the operational properties of the titanium surface.

**Figure 4:**
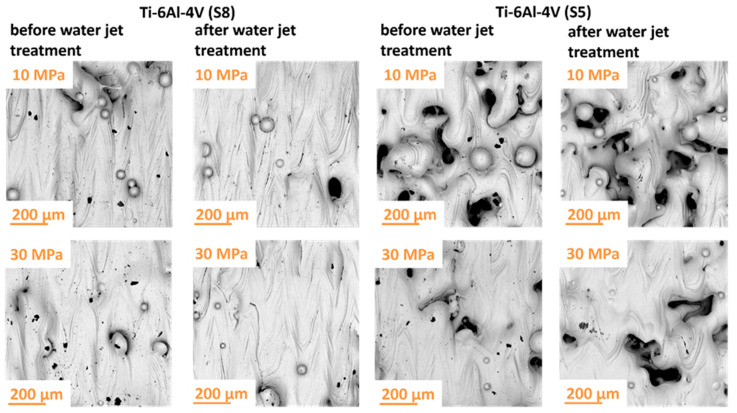
Surface morphology of Ti-6Al-4V titanium samples manufactured according to strategies S5 and S8 before and after water jet treatment.

The samples were treated with a water jet generated by a concentric multi-orifice nozzle. The jet composition consisted of approximately 10% water and 90% air by volume. The processes were conducted at different operating pressures (10 MPa, 20 MPa, and 30 MPa), to assess their impact on the processes’ effectiveness. Figure 4 shows images of selected sample surfaces before and after treatment with the water jet.

**Figure 5:**
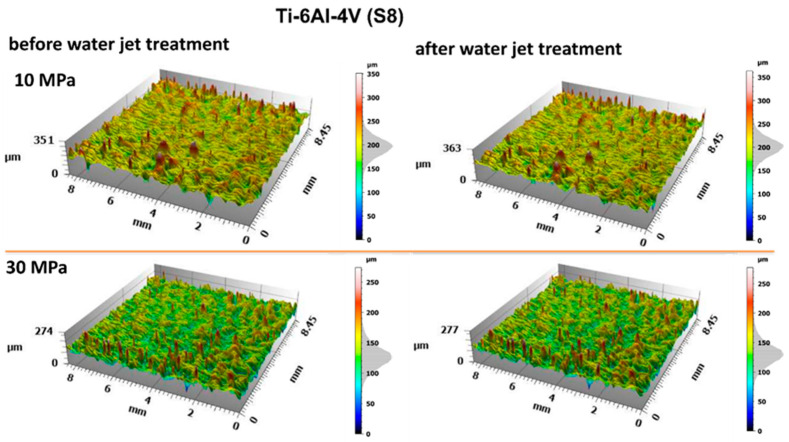
Surface topography of Ti-6Al-4V (S8) before and after water jet treatment at operating pressures of 10 MPa and 30 MPa.

**Figure 6:**
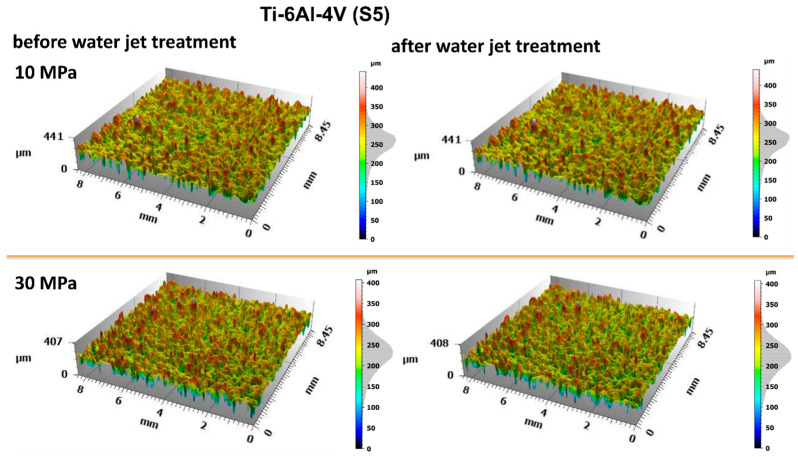
Surface topography of Ti-6Al-4V (S5) before and after water jet treatment at operating pressures of 10 MPa and 30 MPa.

Analysis of the surface morphology of the materials before and after the treatment process revealed no significant changes in their microscopic structure. The use of a water jet, even at elevated pressure levels, primarily resulted in the effective removal of surface contaminants without observable destructive effects on the base material. The increased process efficiency with rising water jet pressure indicates a correlation between the intensity of the process and the effectiveness of contaminant removal. The obtained results suggest that water jet cleaning can be an effective method for contaminant removal while preserving the structure of the cleaned materials at higher pressures. These conclusions are supported by surface topography analyses presented in Figure 5 and Figure 6.

**Figure 7:**
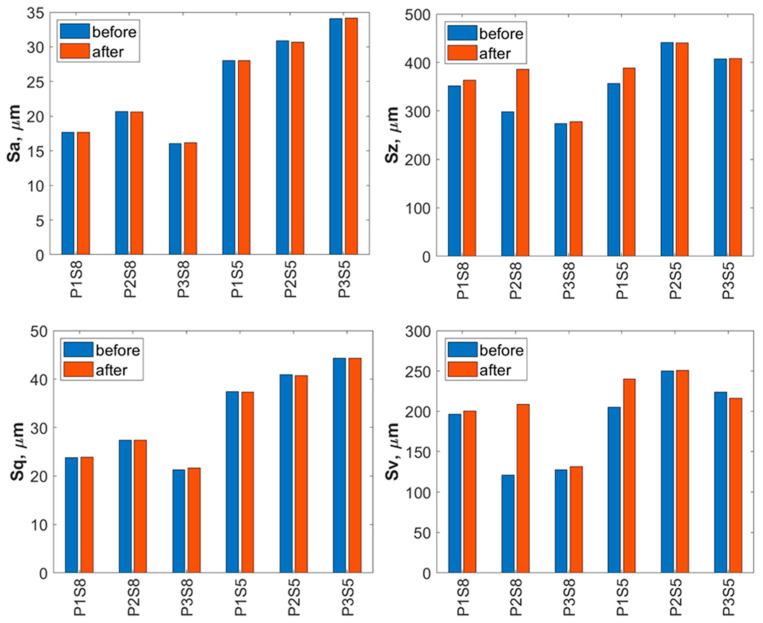
Basic surface parameters of Ti-6Al-4V (S8) and Ti-6Al-4V (S5) before and after water jet treatment (P1—10 MPa, P2—20 MPa, P3—30 MPa).

The obtained surface geometries of the titanium samples reveal relatively uniformly distributed micron-scale irregularities, present both before and after water jet treatment. For a more detailed evaluation of the surface geometric parameters (SGPs), several fundamental amplitude parameters were analyzed. The analysis of individual surface parameters presented in Figure 7 showed no significant changes following water jet treatment.

The results show that the treatment with a water jet did not have a significant effect on the arithmetical mean height (Sa) of the surface. The Sa values for titanium produced using strategy S5 were up to twice as high as those for titanium produced using strategy S8, which is relevant in terms of the surface’s resistance to wear.

The Sq parameter also showed no significant influence of water jet treatment on the analyzed titanium surfaces. The analysis of the maximum height difference (Sz) between the highest peak and the deepest valley revealed only minor changes after the water jet treatment. Titanium samples manufactured using strategy S5 exhibited greater surface irregularities both before and after the treatment.

Additionally, the analysis of the maximum pit depth (Sv)—defined as the depth of the deepest valleys below the core roughness profile—revealed an increase in this parameter for most of the pressure levels applied during the treatment process. Higher Sv values were observed for titanium samples produced using the S5 strategy.

### 3.3. Characteristics of the Surface After Abrasive Water Jet Treatment

**Figure 8:**
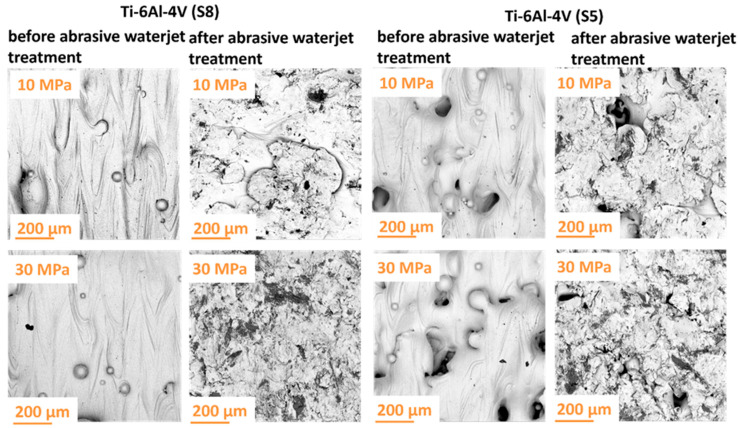
Surface morphology of Ti-6Al-4V titanium samples manufactured according to strategies S5 and S8 before and after abrasive water jet treatment.

The surface quality of materials treated with a high-pressure abrasive water jet is largely influenced by the type of solid particles added to the jet. In the investigated case, quartz sand grains played the dominant role in shaping the surface through repeated particle impacts. These dynamic interactions imparted the treated material with desirable aesthetic and functional properties. Selected surface views of the samples, both before and after abrasive water jet cleaning, are presented in Figure 8.

Based on the obtained results, it can be concluded that the pressure of the abrasive water jet is a key factor determining the effectiveness of the treatment process. The analysis of the surface characteristics of the samples demonstrated that the high velocity of the quartz sand abrasive particles significantly influenced the removal of surface contaminants from the titanium samples. This high particle velocity facilitated the effective removal of surface defects such as unmelted or partially melted powder particles that had adhered to the surface as a result of spatter or thermal diffusion effects. This phenomenon highlights the efficiency of abrasive water jet processing. Consequently, this technique may be particularly advantageous in applications where additional surface finishing is critical.

**Figure 9:**
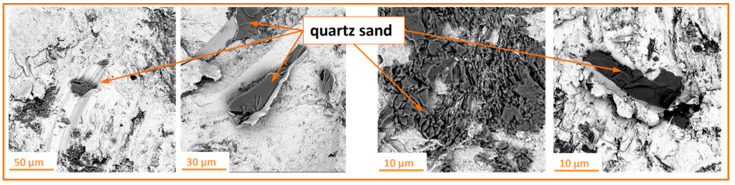
Example of quartz sand particles embedded in the material (Ti-6Al-4V titanium) processed by high-pressure abrasive water jet machining.

During abrasive water jet surface treatment, there is a risk of abrasive particles becoming embedded in the processed material, as also observed by Rauch et al. [29]. An example of such an embedded particle is shown in Figure 9.

Quartz sand grains are characterized by relatively low strength, which causes them to easily fracture during machining, revealing sharp edges. As a result, a significant amount of finer abrasive particles with irregular shapes is generated from the initially rounded grains. This phenomenon is particularly hazardous because an abrasive particle embedded in the material can cause scratches on mating components, adversely affecting their performance and durability. Moreover, an embedded particle becomes a potential corrosion site, which may accelerate the degradation of the internal structure of the components. Therefore, it is crucial to carefully monitor and minimize this risk during machining to ensure improved quality and extended service life of the components.

**Figure 10:**
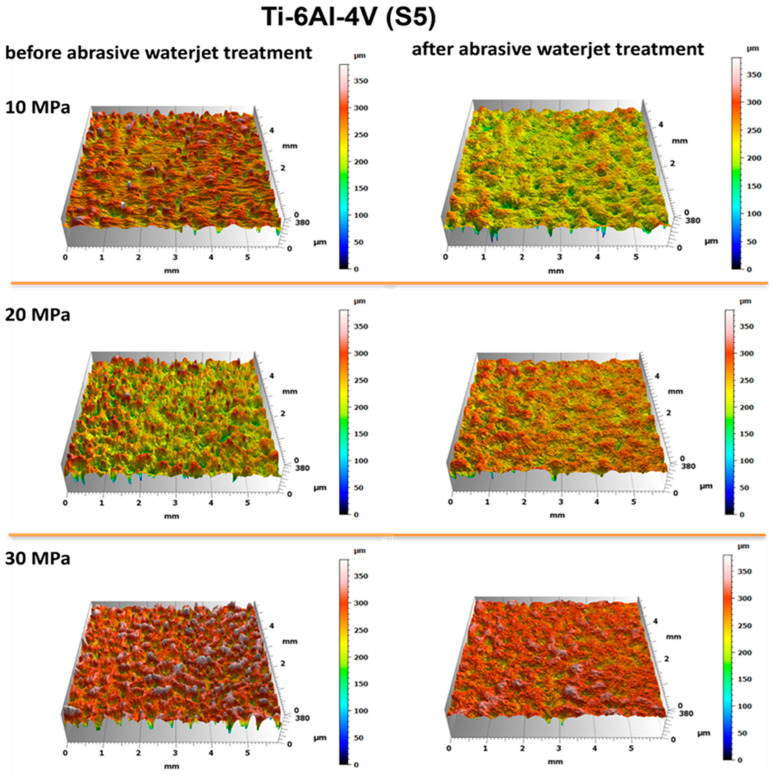
Surface topography of Ti-6Al-4V (S5) before and after abrasive water jet treatment at operating pressures of 10 MPa, 20 MPa, and 30 MPa.

**Figure 11:**
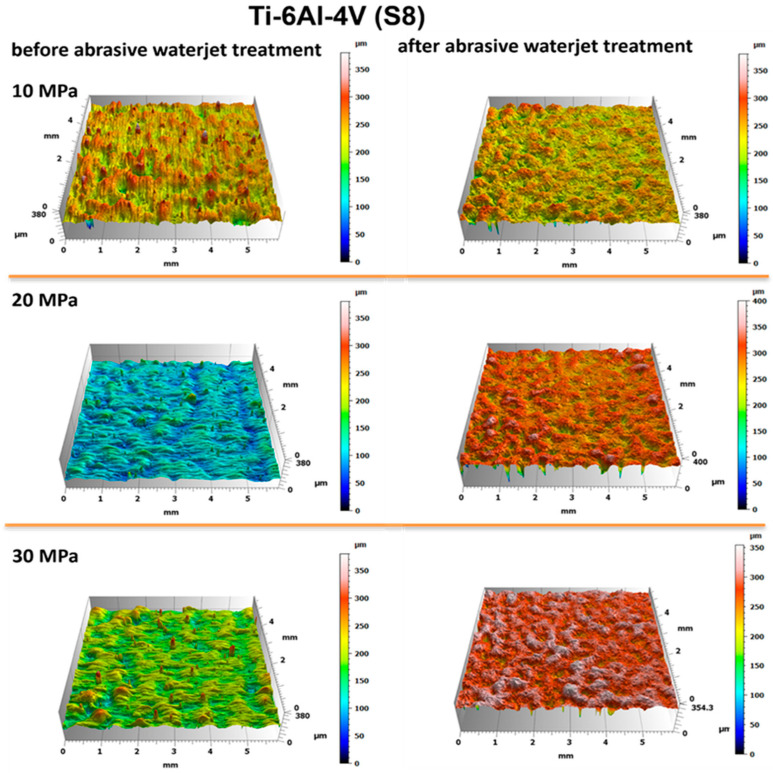
Surface topography of Ti-6Al-4V (S8) before and after abrasive water jet treatment at operating pressures of 10 MPa, 20 MPa, and 30 MPa.

The evaluation of the surface condition of titanium samples treated with an abrasive water jet was conducted by assessing the surface geometric parameters (SGPs) according to the previously described methodology. Figure 10 and Figure 11 present the surface topographies before and after abrasive water jet treatment for Ti-6Al-4V (S5) and Ti-6Al-4V (S8), respectively.

Analysis of the obtained surface topography images of titanium treated with an abrasive water jet demonstrated that the decisive factor affecting surface quality is the jet pressure. The topographies presented in Figure 10 and Figure 11 reveal a developed surface morphology with a uniform distribution of micron-scale irregularities, exhibiting consistent shapes after abrasive water jet treatment. This effect is a fundamental prerequisite for the durable adhesion of coatings to the treated surfaces.

**Figure 12:**
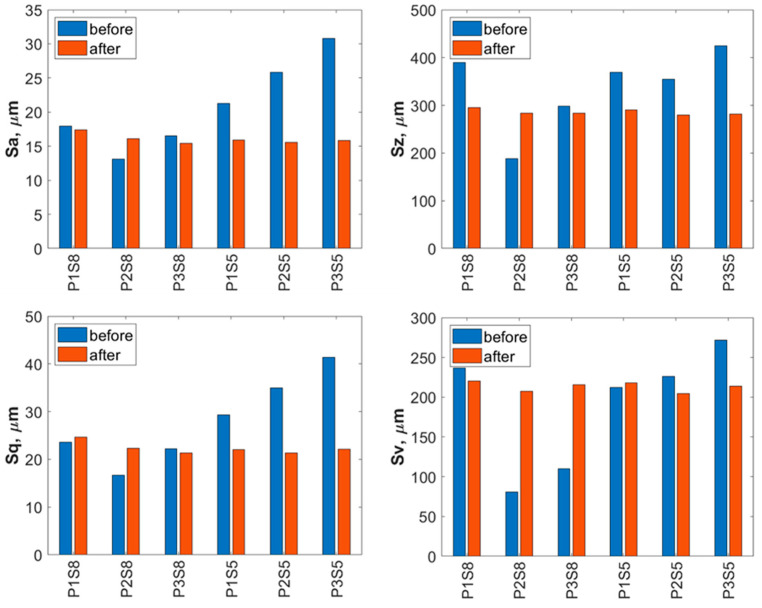
Basic surface parameters of Ti-6Al-4V (S8) and Ti-6Al-4V (S5) before and after abrasive water jet treatment (P1—10 MPa, P2—20 MPa, P3—30 MPa).

A detailed evaluation of the surface geometric parameters (SGPs) was carried out using the basic parameters presented in Figure 12. The analysis of the obtained SGPs revealed that the values of key surface quality indicators changed as a result of the treatment. A lower Sa value typically indicates a smoother surface, which translates into improved tribological properties (reduced friction and wear). A surface characterized by a low Sa parameter may better withstand mechanical loads, thereby enhancing its durability and resistance to damage. The most favorable reduction in the Sa parameter was achieved for the Ti-6Al-4V sample manufactured using the S5 strategy and treated with the highest operating pressure, resulting in a reduction ranging from 25% at a jet pressure of 10 MPa to 50% at 30 MPa. As a result of the treatment process, a reduction in the Sa parameter was achieved—from 25% at a jet pressure of 10 MPa to 50% at a jet pressure of 30 MPa.

The lower Sz values obtained after abrasive water jet treatment, particularly for titanium manufactured using the S5 strategy, indicate a smoother surface (Figure 10). This is clearly illustrated in Figure 12, where titanium samples fabricated using the S5 strategy exhibited a reduction in the Sz parameter of approximately 21% at a jet pressure of 10 MPa, and up to 35% at 30 MPa. In contrast, for the titanium sample produced using the S8 strategy and treated with an abrasive water jet (AWJ) at 20 MPa, an increase in the Sz parameter of about 51% was observed. An increase in Sz was also recorded for this sample after treatment with a pure water jet. However, for the majority of the examined samples, a decrease in the Sz value was achieved.

The lower values of the Sq parameter obtained for most samples after abrasive water jet treatment indicate smoother surfaces, which is advantageous in applications requiring low friction and minimal wear. Surfaces with low Sq values are less susceptible to mechanical damage and can retain their functional properties for a longer time, thereby improving their durability. Similar to the Sa and Sz parameters, the lowest Sq values were observed for titanium samples produced using the S5 strategy. For these S5 samples, Sq values showed a reduction in the root mean square height of the surface profile by approximately 14% at a jet pressure of 10 MPa, and by about 46% at a pressure of 30 MPa.

The analysis of the average pit depth (Sv) for Ti-6Al-4V samples manufactured using the S8 strategy showed that this parameter increased by approximately 200% after abrasive water jet treatment at a pressure of 20 MPa, and up to about 250% at 30 MPa. An increase in the value of the Sv parameter indicates the presence of deeper surface indentations, which translates into higher surface roughness and may negatively affect the aesthetic appearance of the product. Moreover, an increase in the Sv parameter impacts tribological properties, such as friction, which can lead to faster wear of components. However, deeper valleys can retain lubricant more effectively, positively influencing the lubrication of mechanical elements. In certain coating applications, increased surface indentations may also enhance adhesion. Although tribological testing was not performed in this study, it has been reported in the literature that deeper valleys on the surface may facilitate lubricant retention and improve lubrication efficiency under certain conditions [33].

### 3.4. Characteristics of the Surface After Water–Ice Jet Treatment

**Figure 13:**
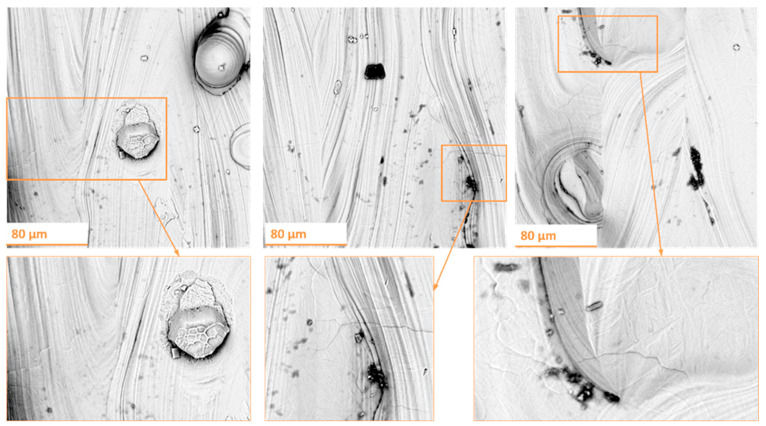
Characterization of surface damage in Ti-6Al-4V titanium alloy subjected to high-pressure water–ice jet machining.

The interaction of the water–ice jet with the surface of the treatment component often manifests as the widening of surface cracks, which leads to an intensified erosion effect. When dry ice (CO_2_) particles impact the surface, rapid sublimation occurs, resulting in a sudden increase in hydrodynamic pressure at the collision site. This phenomenon significantly affects the properties of the processed material. Surface analysis of titanium samples after water–ice jet treatment revealed partial removal of unmelted powder particles and microcracks in the surface layers, as illustrated in Figure 13.

The observed defects may negatively affect the mechanical integrity of the material, highlighting the need for strict control of machining parameters to minimize the formation of such damage. However, the partial removal of unmelted powder particles may have a positive impact on the functional properties of titanium in subsequent applications.

**Figure 14:**
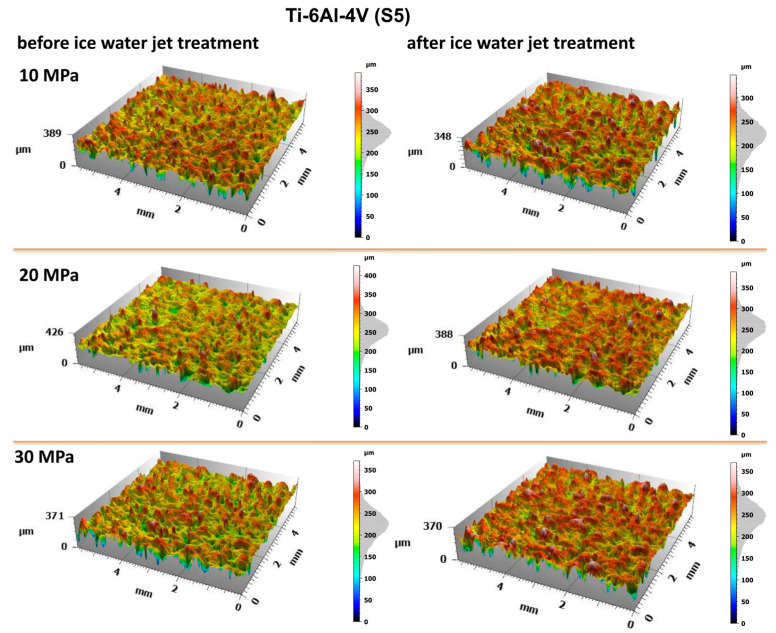
Surface topography of Ti-6Al-4V (S5) before and after water–ice jet treatment at operating pressures of 10 MPa, 20 MPa, and 30 MPa.

**Figure 15:**
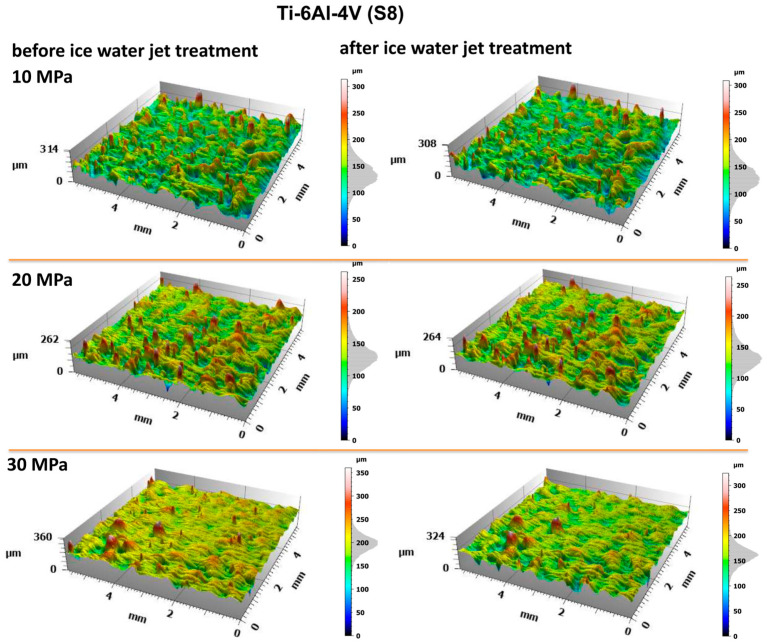
Surface topography of Ti-6Al-4V (S8) before and after water–ice jet treatment at operating pressures of 10 MPa, 20 MPa, and 30 MPa.

Figure 14 and Figure 15 show the surface topography before and after water–ice jet treatment for Ti-6Al-4V (S5) and Ti-6Al-4V (S8), respectively. The analysis revealed no significant changes in the microscopic structure of the samples. The use of the water–ice jet at higher pressures resulted in the removal of certain contaminants, in some cases leading to the elimination of unmelted powder particles and microcracks in the surface layers. The results indicate that water jet cleaning can be effective in removing surface contaminants without compromising the internal structure of the cleaned materials, particularly when higher pressures are applied.

**Figure 16:**
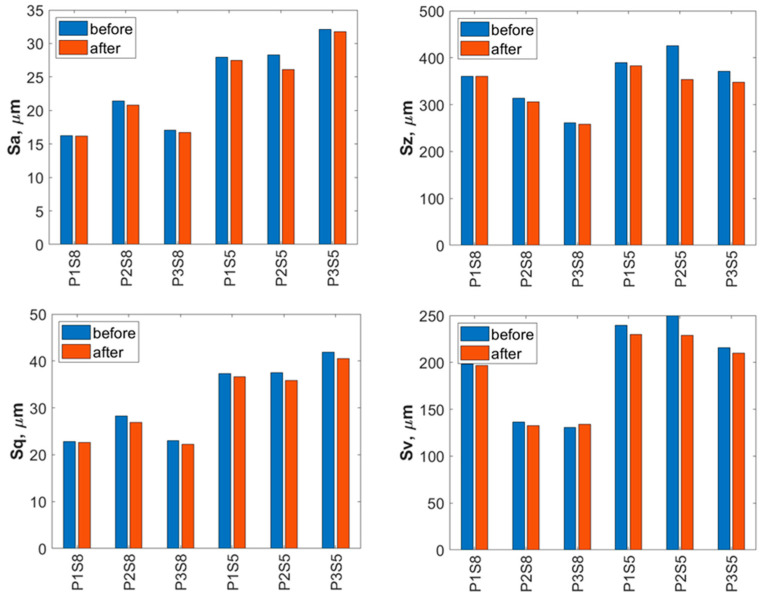
Basic surface parameters of Ti-6Al-4V (S8) and Ti-6Al-4V (S5) before and after water–ice jet treatment (P1—10 MPa, P2—20 MPa, P3—30 MPa).

The values of the basic surface geometric parameters (SGPs) are presented in Figure 16. The analysis of the basic surface roughness parameters before and after treatment with the water–ice jet showed that this process did not have a significant effect on the arithmetic mean surface height (Sa). In some cases, a slight decrease in the Sa parameter—up to a maximum of 8%—was observed. The Sa values for titanium with a relative density corresponding to strategy S5 were up to twice as high as those for titanium with the S8 relative density, which is relevant in terms of surface wear resistance.

The analysis of the maximum height difference between the highest and lowest points of the surface (Sz) also revealed only minor changes after water–ice jet treatment. The greatest reduction in the Sz parameter (approximately 17%) was obtained for the Ti-6Al-4V sample produced using the S5 strategy and treated at a pressure of 20 MPa. The interaction of the water–ice jet with the analyzed titanium surfaces resulted in a slight decrease in the root mean square height (Sq) by a few percent. The analysis of the maximum pit depth (Sv), which reflects the depth of valleys below the core roughness profile, showed a reduction of 2–8% in Sv values for titanium samples manufactured using S5 strategy.

## 4. Conclusions

The analysis of surface microstructures demonstrated that each applied method has its own characteristics and applications. When examining the influence of additive manufacturing technology on the initial surface quality, the following observations were made:

1. The additive manufacturing strategy has a significant impact on the surface quality of Ti-6Al-4V samples.
2. Strategies such as S5, which involve high scanning speeds and low laser power, result in numerous surface defects (e.g., pores, unmelted powder particles) and high surface roughness (Sa ≈ 30 μm).
3. In contrast, the S8 strategy, performed in a stable conduction mode, produced surfaces with fewer defects and lower roughness (Sa ≈ 17 μm).

The use of high-pressure water jet treatment did not have a significant effect on the surface geometric parameters (SGPs). Greater effectiveness was observed with abrasive water jet treatment, leading to the following conclusions:

1. Abrasive water jet treatment technology, particularly at higher pressures (30 MPa), is effective in removing surface defects and residuals from the additive manufacturing process (e.g., unmelted powder particles).
2. This process results in a noticeable reduction in the surface roughness parameter (Sa), indicating surface smoothing and potentially improved functional properties.
3. The uniform surface morphology achieved after this treatment promotes better adhesion of protective and functional coatings.
4. However, there is a risk of entrapped abrasive particles (e.g., quartz sand) remaining in the material, which may lead to microdamage, corrosion initiation, and reduced service life.

The application of water–ice jet treatment shows promising results in the context of titanium elements post-processing, combining the effect of low process temperature with high material removal efficiency, making this technique an interesting alternative to conventional machining methods. Partial elimination of unmelted powder particles may positively influence the operational properties of the additively manufactured elements, enhancing its potential for further engineering applications.

When comparing our findings with alternative post-processing methods reported in the literature, it is evident that techniques such as laser polishing [9,14,18], chemical polishing [16], and shot peening [23] may achieve lower roughness values or enhance other functional properties. However, abrasive water jet treatment remains advantageous due to its environmentally friendly character, efficiency in removing unmelted powder particles, and suitability for large, complex components.

Studies have confirmed that different processing methods for Ti-6Al-4V alloy have specific advantages. The final selection of the method should depend on the particular application requirements and the desired properties of the finished products. Future work will focus on further developing effective combinations of these methods, as well as their optimization or modification to achieve satisfactory titanium surface quality.

## Figures and Tables

**Table 1:** Samples manufacturing strategies.

Parameter	Strategy Symbol	
	S5	S8
Laser power, P [W]	180	250
Scanning speed, v [mm/s]	1200	
Hatching distance, h [mm]	0.1	
Layer thickness, t [mm]	0.03	
Volumetric energy density, E_v_ [J/mm^3^]	50	70

**Table 2:** Chemical composition of Ti-6Al-4V titanium alloy powder.

	Ti	Al	V	Fe	O	C	N	H
**Ti-6Al-4V**	Balance	6.00	4.00	≤0.25	≤0.13	≤0.08	≤0.03	≤0.012

**Table 3:** Specification of WEMAA hydro monitor.

Parameter	Values
Maximum pressure [MPa]	50
Pump capacity [dm^3^/s]	0.25
Engine power [kW]	15

**Table 4:** Specification of abrasive materials used in the surface treatment processes.

Parameter	Material	
	Quartz Sand	Dry Ice Granules
Grain size [mm]	0.5–1.5	3–16
Density [kg/m^3^]	2650	1500
Mohs hardness	5–7	2–3

## Data Availability

The raw data supporting the conclusions of this article will be made available by the authors on request.
